# Comparison of Minimalist Footwear Strategies for Simulating Barefoot Running: A Randomized Crossover Study

**DOI:** 10.1371/journal.pone.0125880

**Published:** 2015-05-26

**Authors:** Karsten Hollander, Andreas Argubi-Wollesen, Rüdiger Reer, Astrid Zech

**Affiliations:** 1 Department of Sports and Exercise Medicine, Institute of Human Movement Science, University of Hamburg, Hamburg, Germany; 2 Institute of Human Movement Science, University of Hamburg, Hamburg, Germany; 3 Department of Exercise Physiology, Institute of Sports Science, Friedrich Schiller University of Jena, Jena, Germany

## Abstract

Possible benefits of barefoot running have been widely discussed in recent years. Uncertainty exists about which footwear strategy adequately simulates barefoot running kinematics. The objective of this study was to investigate the effects of athletic footwear with different minimalist strategies on running kinematics. Thirty-five distance runners (22 males, 13 females, 27.9 ± 6.2 years, 179.2 ± 8.4 cm, 73.4 ± 12.1 kg, 24.9 ± 10.9 km.week^-1^) performed a treadmill protocol at three running velocities (2.22, 2.78 and 3.33 m.s^-1^) using four footwear conditions: barefoot, uncushioned minimalist shoes, cushioned minimalist shoes, and standard running shoes. 3D kinematic analysis was performed to determine ankle and knee angles at initial foot-ground contact, rate of rear-foot strikes, stride frequency and step length. Ankle angle at foot strike, step length and stride frequency were significantly influenced by footwear conditions (p<0.001) at all running velocities. Posthoc pairwise comparisons showed significant differences (p<0.001) between running barefoot and all shod situations as well as between the uncushioned minimalistic shoe and both cushioned shoe conditions. The rate of rear-foot strikes was lowest during barefoot running (58.6% at 3.33 m.s^-1^), followed by running with uncushioned minimalist shoes (62.9%), cushioned minimalist (88.6%) and standard shoes (94.3%). Aside from showing the influence of shod conditions on running kinematics, this study helps to elucidate differences between footwear marked as minimalist shoes and their ability to mimic barefoot running adequately. These findings have implications on the use of footwear applied in future research debating the topic of barefoot or minimalist shoe running.

## Introduction

The last few years, barefoot and barefoot-like running has been widely discussed as a natural alternative to traditional shoe running in recreational sports [1,2]. The long-believed benefits of stable and cushioned running shoes are questioned by findings that show lower prevalence of foot disorders [3,4], improved running economy [5,6] and lower impact forces in barefoot runners [5,7,8]. These effects are probably due to alterations in lower extremity running biomechanics. Numerous studies [5–8] have shown a higher rate of rear-foot strikes (RFS) during running with shoes whereas barefoot running produces more forefoot strikes during initial ground contact. According to Lieberman et al. [7], this is mainly caused by cushioning of the heel, which allows “a runner to rear-foot strike comfortably” by reducing peak ground reaction forces. However, forefoot running patterns are not only a result of missing shoe cushioning. They also occur more frequently at increased running speeds, are influenced by the running surface, and are dependent on individual habituation [7,9–11]. Hence, the reported kinematic and kinetic characteristics of barefoot running [1,7,8] are more likely due to a more plantarflexed footstrike than to the footwear condition [12]. Although the forefoot ground contact and lower impact forces are also often believed to be associated with a reduced injury risk, no conclusive evidence exists on the influence of regular barefoot running on lower extremity injury rates [9,13–16].

Running with bare feet is sometimes restricted by hard and unsafe ground conditions or low temperatures. In recent years, the development of barefoot-like footwear with reduced cushioning and/or high flexibility has gained increasing attention among numerous manufactures. In the literature, shoes with minimal cushioning and weight, and/or increased sole flexibility are typically referred to as “minimalist shoes”, “lightweight shoes” or “barefoot shoes”. The effectiveness of minimalist footwear for simulating barefoot running is mostly unclear due to inconsistent findings in the literature. Squadrone and Gallozzi [17] found similar ankle angles at initial foot ground contact during barefoot running and running with uncushioned minimalist shoes. Both conditions were significantly different from standard shoe running. Bonacci et al. [18] reported significant differences between cushioned minimalist shoes with ultraflexible soles and barefoot condition in knee flexion and ankle dorsiflexion during initial ground contact. Taking the discrepant findings into account, it seems reasonable that shoe cushioning plays an important role in the simulation of barefoot running. First data on the influence of different midsole thicknesses compared to no cushioning (barefoot) were previously shown regarding joint stiffness, vertical ground reaction force and strike index [19]. However to our knowledge, no study has yet compared the effects of minimalist shoes with different characteristics regarding cushioning and weight on running kinematics in one study protocol. The differentiation between these effects may help to understand the relevant factors of barefoot running simulation.

The objective of this study was to determine the influence of shoe cushioning and flexibility on treadmill running ankle and knee kinematics in habitual shod runners. Two varying minimalist shoe models of different cushioning were compared with barefoot and standard footwear conditions at three running speeds. Considering previous findings, we hypothesize that kinematics during running with uncushioned minimalist shoes are closer to barefoot conditions than cushioned minimalist shoes.

## Methods

This study had a randomized crossover design and took place in a University Biomechanics Laboratory. Ethical approval for the study was obtained from the ethics committee of the medical association Hamburg (protocol no. PV4271). Prior to the study all participants provided their written informed consent to participate in this study. The study followed the principles of the Helsinki Declaration.

For inclusion, participants had to be recreational runners, running at least 12 km per week, between 18 and 45 years of age and free of orthopedic, neurological or musculoskeletal disorders for the past six months. Participants were not allowed to have any experience with minimalist running shoes. Both, habitually forefoot and rear-foot strikers were considered for participation.

**Fig 1:**
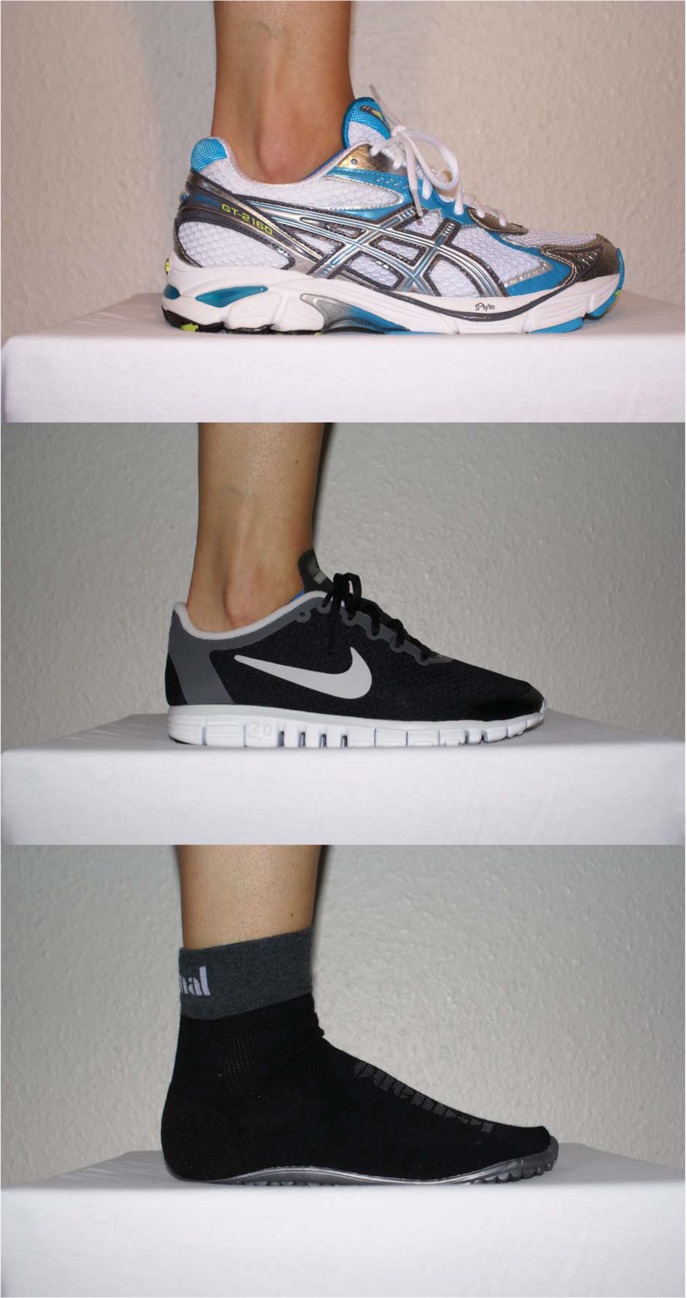
Shoe conditions. (top image = Asics GT-2160, center image = Nike free 3.0, lower image = Leguano).

In this study, four different conditions were applied in random order: barefoot running, standard running shoe running, cushioned minimalist shoe running and uncushioned minimalist running shoe running (Fig 1). The order was counterbalanced between the first thirty-two participants and partly balanced between the last three participants. All shoes were commercially available. An Asics GT-2160 (ASICS, Kobe, Japan) was used as standard running footwear. It has an ethylene-vinyl acetate midsole, an arch support, 12 mm heel-forefoot offset and a weight of 314 g (woman’s shoe, US size 6.5). As a representative of cushioned minimalist footwear, a Nike Free 3.0 (NIKE, Beaverton, OR, USA) with a 4 mm heel-forefoot offset, no arch support and a weight of 189 g was used. A Leguano (LEGUANO, St. Augustin, Germany) was used for uncushioned minimalist footwear. It has a polyvinyl chloride midsole, 0 mm heel-forefoot offset, no arch support and a weight of 137 g. Cushioning properties of shoes were measured using a drop tester, designed according to the American Society for Testing and Material (ASTM’s) "Standard Test Method for Shock Attenuating Properties of Materials Systems for Athletic Footwear" (F1976, ASTM International, West Conshohocken, PA, USA). An indenter of 35 mm diameter with a load cell completed 10 impacts on the heel of one shoe of each footwear condition (US size 6.5). For the standard running shoe the peak impact force was 750 N with a maximum impact depth of 7.70 mm. The cushioned minimalist shoe produced a peak impact force of 845 N and a maximum impact depth of 7.49 mm. Peak impact force of the uncushioned minimalist shoe was 2200 N and the maximum impact depth 1.85 mm.

The primary outcome was ankle angle at footstrike. Secondary outcomes were knee angle at footstrike, rate of rear-foot strike (RFS), step length and stride frequency. Kinematic analysis was performed using a three-dimensional 8-camera infrared motion analysis system operating at 200 Hz (VICON, Oxford, UK). The cameras were placed around a treadmill (Ergo-Fit TRAC 4000, ERGO-FIT GmbH & Co. KG, Pirmasens, GERMANY) for data collection with minimized marker occlusions. According to the Plug-in-Gait model (VICON, Oxford, UK), sixteen retro-reflective markers (14 mm diameter) were located bilaterally at anatomical bony landmarks of the pelvis, thigh, knee, shank, ankle, and foot as used in a prior study [20]. To enable calculation of knee and ankle joint angles, the following anthropometric measures were obtained: bilateral leg length, knee width, ankle width, height, and body mass.

After randomization of footwear conditions, participants ran each condition at three different velocities (*v*_*1*_ = 2.22 m^.^s^-1^, *v*_*2*_ = 2.78 m^.^s^-1^, *v*_*3*_ = 3.33 m^.^s^-1^). All markers remained on the identical position, except for the foot markers. They were adjusted for each condition on the surface of the shoe in reference to the foot. Calcaneal and second metatarsal marker were kept at the same height and level of the shoe. The same distance to the ground was determined by the use of a caliper. Additionally, standing calibrations were taken separately for each footwear condition. This individual capture of calibration trials were used to create a biomechanical model of the lower body (Plug-In-Gait).

An accommodation to the treadmill and a warm-up period by walking in a self-selected velocity was conducted. Participants were asked to indicate readiness and the treadmill was accelerated to 2.22 m^.^s^-1^ with a rate of 0.2 m^.^s^-2^. Thirty seconds afterwards, data recording started for fifteen seconds over two consecutive sessions during each trial. The second recording was taken as a backup. After data collection for the first velocity, participants were given a one-minute rest. The same procedure was applied for 2.78 m^.^s^-1^ and 3.33 m^.^s^-1^. Then subsequently, the footwear condition changed according to the randomization protocol and the test procedure was repeated equally for each condition.

Kinematic data was filtered using a Woltring filtering routine (mean square error = 15). All data processing was done using Vicon Nexus 1.7.1 and Polygon 3.5.1 (VICON, Oxford, UK). Footstrike was defined when the vertical velocity of the distal heel marker changed from negative to positive. This method was recently described as the most valid and reliable method for the kinematic identification of foot strike [21].

Outcomes of interests were ankle and knee angles in the sagittal plane during the phase of initial ground contact. Ankle dorsiflexion and knee flexion angles were recorded during the whole gait cycle and analyzed only during the last 10% of gait cycle (before the identified ground contact). The time interval was utilized in order to address the sources of error that occur when the velocity of the distal heel marker is used to identify the initial ground during different footwear conditions with and without cushioning. Gait cycle data were compared to neutral standing position. A virtual biomechanical model was developed for each subject and condition. Rate of RFS was determined visually by examination of a lateral high-speed video independently by two investigators.

Ankle and knee kinematics, step length and stride frequency were analyzed for ten consecutive gait cycles. Individual knee and ankle angle kinematic data for each leg were processed using Matlab software (Mathworks, Natick, MA, USA). Trials were normalized to 100% of gait cycle. The Vicon motion capture system is a reliable tool for the analysis of gait kinematics [22,23].

To determine differences between shoe conditions, we calculated mixed models [24] for interesting metric dependent variables ankle and knee angle at footstrike, step length, and stride frequency. To adjust for the cluster structure, participants were included as a random factor. The interesting main effect of shoe condition was included as a fixed effect as well as the factors running velocity and leg side. Tentatively, two-way interactions between Shoe×Sex and Shoe×Side (left/right) were added and kept in all models if significant. Furthermore, a Bonferroni post hoc test was conducted between shoe conditions. Cohen's d was calculated by using the difference between the means divided by the pooled standard deviation. A generalized estimating equation model for a repeated measures logistic regression was calculated for the dichotomous variable “rate of RFS”. For shoe comparisons odds ratios are presented. The SPSS statistical package Version 21 (SPSS, Armonk, NY, USA) was used for all statistical procedures.

## Results

Thirty-five recreational distance runners took part in the study (22 males, 13 females, age = 27.9 ± 6.2 years, height = 179,2 ± 8,4 cm, mass 73.4 ± 12.1 kg, mileage = 24.9 ± 10.9 km^.^week^-1^). All participants were habitual shod runners who were used to treadmill running. Two participants were habitual forefoot runners.

Kinematic parameters for all running conditions are shown in Table 1. Footwear conditions and running velocity significantly (p<0.001) influenced ankle angles, stride frequency and step length (Table 2). Ankle angles differed with statistical significance (p<0.001) between all shoe conditions for each velocity except for comparison of cushioned minimalist and standard shoe condition (p = 0.674) (Table 3). Running barefoot reduced the dorsiflexion by 1.73° (95% CI 0.99°;2.48°) compared to uncushioned minimalist shoes, 5.52° (95% CI 4.77°;6.27°) compared to cushioned minimalist shoes and 5.68° (95% CI 4.96°,6.47°) compared to standard shoes. The uncushioned minimalist running condition produced a 3.78° (95% CI 3.04°;4.53°) lower dorsiflexion during foot landing than the cushioned minimalist running. Additionally, running velocity (p<0.001), body weight (p<0.05) and weekly mileage (p<0.05) significantly influenced ankle angle at footstrike (Table 2). There was no statistically significant effect of shoe conditions on the knee angle at footstrike (p = 0.239). Effects on the knee angle at footstrike were found for velocity (p<0.001) and sex (p<0.05). Females produced higher knee angels at footstrike compared to males.

**Table 1:** Group mean (SD) temporal-spatial and kinematic parameters for 2.22, 2.78 and 3.33 m^.^s^-1^.

	Barefoot	Uncushioned minimalist shoe	Cushioned minimalist shoe	Standard running shoe
**2.22 m** ^.^ **s** ^**-1**^				
Ankle angle at footstrike (°)	6.90 (5.95)	8.69 (6.12)	11.66 (4.88)	11.14 (4.16)
Knee angle at footstrike (°)	10.77 (5.26)	10.53 (4.71)	10.07 (4.24)	10.02 (4.51)
Stride frequency (steps^.^minute^-1^)	160.87 (5.46)	158.14 (6.06)	155.70 (7.78)	154.47 (5.14)
Step length (cm)	82.98 (2.82)	84.44 (3.25)	85.80 (3.83)	86.41 (2.92)
Rate of rear-foot strikes (%)	62.9	74.3	90.0	94.3
**2.78 m** ^.^ **s** ^**-1**^				
Ankle angle at footstrike (°)	5.70 (6.46)	7.39 (6.19)	11.57 (4.74)	11.33 (4.24)
Knee angle at footstrike (°)	9.77 (6.99)	10.83 (4.48)	10.27 (5.26)	10.65 (5.24)
Stride frequency (steps^.^minute^-1^)	167.09 (8.18)	164.36 (7.44)	161.68 (7.52)	158.68 (5.98)
Step length (cm)	99.98 (4.91)	101.61 (4.60)	103.30 (4.85)	105.18 (3.96)
Rate of rear-foot strikes (%)	55.7	68.6	92.9	94.3
**3.33 m** ^.^ **s** ^**-1**^				
Ankle angle at footstrike (°)	4.68 (7.23)	6.40 (6.80)	10.56 (5.23)	11.85 (4.12)
Knee angle at footstrike (°)	12.56 (5.73)	12.52 (5.27)	12.03 (5.16)	11.40 (4.89)
Stride frequency (steps^.^minute^-1^)	174.85 (9.90)	170.80 (8.52)	168.60 (8.43)	164.84 (7.44)
Step length (cm)	114.74 (6.37)	117.38 (5.83)	118.92 (5.93)	118.15 (6.37)
Rate of rear-foot strikes (%)	58.6	62.9	88.6	94.3

SD standard deviation

**Table 2:** Mixed model effects (p-values) for included factors.

	Footwear	Running Velocity	Leg side	Footwear* Velocity
Ankle angle at footstrike (°)	<.001	.001	.699	.026
Knee angle at footstrike (°)	.239	<.001	.157	.285
Stride frequency (steps.minute-1)	<.001	<.001	.611	<.001
Step length (cm)	<.001	<.001	.622	<.001

**Table 3:** Differences (95% CI) and p-values of pairwise comparisons between footwear conditions.

	Ankle angle at footstrike (°)			Knee angle at footstrike (°)			Stride frequency (steps.minute-1)			Step length (cm)		
	Diff. (CI)	P-value	Cohen’s d	Diff. (CI)	P-value	Cohen’s d	Diff. (CI)	P-value	Cohen’s d	Diff. (CI)	P-value	Cohen’s d
Barefoot vs Standard running shoe	-5.68 (-6.426; -4.935)	<.001	-1.032	.346 (-.319; 1.010)	.308	.062	8.275 (7.624; 8.926)	<.001	.945	-5,156 (-5,586;-4,726)	<.001	-.357
Barefoot vs Uncushioned minimalist shoe	-1.73 (-2.479;-.988)	<.001	-.946	-.258 (-.923; .407)	.446	.045	3.171 (2.520; 3.823)	<.001	.589	-1,910 (-2,340; -1,480)	<.001	-.244
Barefoot vs cushioned minimalist shoe	-5.52 (-6.267;-4.772)	<.001	-.461	.310 (-.357; .976)	.362	-.047	5.613 (4.962; 6.264)	<.001	.337	-3,442 (-3,872; -3,013)	<.001	-.136
Uncushioned minimalist shoe vs cushioned minimalist shoe	-3.79 (-4.534;-3.039)	<.001	-.464	.568 (-.098; 1.235)	.095	.103	2.441 (1.790; 3.093)	<.001	.268	-1,532 (-1,962; -1,103)	<.001	-.107
Uncushioned minimalist shoe vs standard running shoe	-3.95 (-4.692;-3.201)	<.001	-.527	.604 (-.061; 1.268)	.075	.123	5.104 (4.452; 5.755)	<.001	.615	-3,246 (-3,676;-2,816)	<.001	-.222
Cushioned minimalist shoe vs standard running shoe	-.16 (-.908;.587)	.674	-.037	.036 (-.631; .702)	.917	.019	2.662 (2.011; 3.313)	<.001	.317	-1,714 (-2,143; -1,284)	<.001	-.117

The repeated measures logistic regression analysis showed that rate of rear-foot strikes was not significantly influenced by velocity (p = .294), sex (p = .415) or leg side (p = .234). Significantly different RFS were shown for the different footwear conditions (p<.001). During all velocities, the RFS was highest for standard shoe running, followed by cushioned minimalist shoe, uncushioned minimalist shoe and barefoot conditions (Table 1). Statistically significantly different odds ratios were found between barefoot and both cushioned shoe conditions (2.22 m^.^s^-1^ OR = .188 (95% CI: .075, .471) and OR = .103 (95% CI: .033, .314)) as well as between uncushioned minimalist and both cushioned shoe conditions (2.22 m^.^s^-1^ OR = .321 (95% CI: .284, 1.207) and OR = .175 (95% CI: .056, .549)). Running barefoot and with uncushioned minimalist shoes did not differ for the rate of RFS (2.22 m^.^s^-1^ OR = .586 (95% CI: .075, .471)).

Regarding temporal-spatial outcomes, running barefoot, subjects took the smallest steps with the highest stride frequency compared to uncushioned minimalist (p<.001), cushioned minimalist (p<.001) and standard shoes (p<.001). Stride frequency was higher and step length shorter during running with uncushioned minimalist shoes compared to cushioned minimalist shoes (<.001). The standard running shoe condition led to the highest step length and smallest stride frequency. Running velocity also influenced stride frequency and step length significantly (p<0.001) (Table 2).

## Discussion

The objective of this study was to identify minimalist footwear characteristics responsible for the simulation of barefoot running kinematics. In a random and counterbalanced order cushioned and uncushioned minimalist shoes were compared to standard cushioned shoe and barefoot conditions. The study’s hypothesis was that kinematics during running with uncushioned minimalist shoes are closer to barefoot conditions than cushioned minimalist shoes.

In agreement with other studies [20,25,26], we found significant differences in ankle kinematics and step length as well as stride frequency between barefoot running and all shod running conditions. The most remarkable differences were observed between barefoot and cushioned shoe conditions. The main finding of this study was that minimalist shoes differ in their ability to simulate barefoot running. All outcome measures except for the knee angle were significantly different between cushioned and uncushioned minimalist shoes.

Minimalist footwear has been designed in order to replicate barefoot running and is increasingly used by recreational runners [27]. While the impact of barefoot running on biomechanics is widely discussed, it has not yet been defined which shoe characteristics adequately meet the criteria to mimic barefoot running biomechanics. Hence, current minimalist shoe models differ in their cushioning and flexibility characteristics and produce uncertainty regarding the comparability of running in barefoot-simulating footwear and real barefoot running. Our results show that the effectiveness of minimalist footwear for simulating barefoot running kinematics seems to be influenced by the cushioning properties. The findings are in accordance with the findings of Squadrone & Gallozzi [17], who used a minimalist shoe (Vibram five-fingers) similar to the one used in our study (no cushioning, 0 mm heel-forefoot offset). Contrary to our finding, the authors observed no differences in the ankle dorsiflexion angle at foot strike between barefoot and minimalist shoe running. The findings reported by Bonacci et al [18], who used the same cushioned minimalist shoe (Nike Free 3.0), are comparable to our results concerning ankle kinematics. They reported significant differences in knee and ankle kinematics between minimalist shoe and barefoot running conditions. However, the comparability between both studies is further limited due to different populations used. While Squadrone & Gallozzi [17] investigated habitually barefoot runners, Bonacci et al [18] analyzed subjects that were highly trained but habitually shod. Taking these considerations and our results into account, one can say that footwear with less heel-forefoot offset and less cushioning seem to be more capable of replicating barefoot running than shoe models without these characteristics.

In this study, running shod led to increased ankle angles at footstrike compared to barefoot running. These findings are in agreement with several other studies [7,20,26,28]. The lack of differences in knee angles, however, are inconsistent compared to other research [18,25]. This might be explained with the effect of gender on the knee angle shown in this study or the different populations investigated. Our participants were recreational and habitually shod runners. Other studies compared habitually shod and habitually barefoot runners [7], highly trained runners [18], exclusively male runners [25] or runners that were just included when being habitual shod heelstriker [29]. The lower ankle dorsiflexion angles in our study indicate a flatter foot at landing for barefoot and uncushioned shoe running. Hence, it is not surprising that both running conditions significantly decreased the rate of rear-foot strikes among participants. Nevertheless, it should also be noted that during barefoot and minimalist running, the RFS was still present in more than 50% of the participants. The flatter foot placement at initial contact is a typical characteristic of barefoot running [7,28,30]. It is generally believed that this is a common strategy in order to generate lower impact forces during initial ground contact [28]. Our data indicate that the lack of cushioning might be predominantly responsible for this effect. However, it should also be considered that this landing pattern seems to depend on the running surface and speed as well as on the subject [30].

Furthermore, our research showed an increase of stride frequency and a decrease of step length when running barefoot. These findings have been reported in many other studies for healthy adult [9], adolescent [5] and infantile [20,31] populations. They are probably a consequence of a smaller impact force during landing [17] but might also be explained by a more cautious gait due to higher proprioception [32]. It has been previously shown that taking smaller steps reduces the impact force peak and loading rates [33] and may prevent impact-related injuries [34].

Some limitations should be considered in interpretation of findings. First of all, neither participants, nor researchers were masked to the running condition, which may have induced bias towards the benefits of a particular running condition. Nevertheless, no information was given to participants on the study hypothesis. Furthermore, the marker placement on the shoe surface causes the second metatarsal head marker to be slightly more superior compared to the attachment directly on the skin. Other studies [29,35] addressed this problem by cutting windows into the shoe’s upper material or using sandals [36]. We adjusted the superior-inferior position of the heel marker and used separate calibrations for each condition. The most important limitation in this study is the lack of ground reaction force data allowing direct conclusions on running kinetics. Therefore, the discussion of impact forces during landings in this study remains mainly speculative. Our study also lacks the ability to make conclusions about the footwear’s influence on injury risk or prevention. In contrast to the widely discussed beneficial effects of minimalist footwear, two recent studies show first evidence about an increased injury risks due to minimalist footwear training [15,16]. In accordance with other studies [37,38], we conclude that well-powered prospective studies are needed to elucidate relationship between the influence of shoes and running injuries.

## Conclusion

In this study, running kinematics of healthy long distance runners were influenced by footwear and running velocity. Ankle dorsiflexion angles and rate of rear-foot strikes were lowest during the barefoot running condition and increased with augmented cushioning properties of footwear. Running kinematics for uncushioned minimalist shoes were closer to barefoot running kinematics than those of cushioned minimalist shoes. The results indicate that cushioning plays an important role for simulating barefoot running kinematics. These findings have implications on the use of footwear used in future research debating the topic of barefoot or minimalist shoe running.
